# Meetings of Societies

**Published:** 1934

**Authors:** 


### Meetings of Societies

### Bristol Medico-Chirurgical Society

SESSION 1933–34.

The Annual Meeting of the Society was held in the Physiological Lecture Theatre of the University on Wednesday, 10th October, at 8.30 p.m.

Dr. Elwin Harris, senr., was in the Chair, and 104 members were present.

The minutes of the previous meeting were read and, with one amendment, were confirmed.

The President then resigned his Chair to his successor, Professor A. Rendle Short. A vote of thanks to the retiring President was proposed by Dr. J. A. Nixon and seconded by Dr. Corfield, and carried with acclamation, and Dr. Elwin Harris replied.

The President then gave his inaugural address on the "**Origin of the Human Race.**" A vote of thanks to the President for his address was proposed by Mr. T. Carwardine and seconded by Mr. C. Moore, and this was carried with acclamation.

The annual reports of the Hon. Secretary and Hon. Treasurer and of the Editor of the Society's *Journal* were read and adopted, and Mr. H. L. Shepherd and Mr. A. E. Iles were re-elected as Hon. Secretary and Hon. Treasurer respectively.

Mr. Fitzgibbon proposed, and Dr. Phillips seconded, that the nine members elected to the Committee should be :—

Mr. Clifford Moore.

Mr. Duncan Wood.

Dr. A. L. Flemming.

Dr. H. L. Fuller.

Dr. P. S. Martin.

Dr. Vincent Wood.

Dr. Arthur Fells.

Dr. Richard Clarke.

Dr. A. L. Taylor.

and the following three gentlemen were asked to serve on the University Library Sub-Committee : Professor Rendle Short, Dr. George Parker and Dr. J. O. Symes.

### BRISTOL MEDICO-CHIRURGICAL SOCIETY.

### INCOME AND EXPENDITURE ACCOUNT for the year ended 30th September, 1934.

### INCOME.

| | £ | s. | d. | £ | s. | d. |
|---|---|---|---|---|---|---|
| Members' Subscriptions .. | 311 | 17 | 0 | | | |
| Do., outstanding for 1933–1934 .. .. .. .. .. | 31 | 10 | 0 | | | |
| | | | | 343 | 7 | 0 |
| Interest on Bank Deposits .. .. .. | | | | 2 | 8 | 11 |
| Interest on £500 4 per cent. Consolidated Stock, less tax .. .. .. | | | | 15 | 5 | 0 |
| | | | | 361 | 0 | 11 |
| Balance, being excess of Expenditure over Income for the year, carried to Balance Sheet .. .. | | | | 30 | 7 | 4 |
| | | | | £391 | 8 | 3 |

### EXPENDITURE.

| | £ | s. | d. | £ | s. | d. | £ | s. | d. |
|---|---|---|---|---|---|---|---|---|---|
| Grant to *The Bristol Medico-Chirurgical Journal* .. .. .. .. | | | | | | | 319 | 19 | 4 |
| Librarian—Honorarium .. .. .. .. | | | | | | | 15 | 0 | 0 |
| Miscellaneous Expenses— | | | | | | | | | |
| Goodway–Refreshments | | | | 16 | 16 | 0 | | | |
| Postages and Sundries | | | | 14 | 3 | 2 | | | |
| Printing and Stationery | | | | 9 | 6 | 9 | | | |
| Audit Fee .. .. .. .. | | | | 2 | 2 | 0 | | | |
| Lantern and Cinematograph at Meetings .. | | | | 4 | 4 | 0 | | | |
| Lambert—Upholstery .. | | | | 3 | 17 | 6 | | | |
| University Marshal .. | | | | 1 | 0 | 0 | | | |
| Lewis's Medical and Scientific Library— | | | | | | | | | |
| Subscription | 4 | 4 | 0 | | | | | | |
| Postages .. | 0 | 15 | 6 | | | | | | |
| | | | | 4 | 19 | 6 | | | |
| | | | | | | | 56 | 8 | 11 |
| | | | | | | | £391 | 8 | 3 |

### BALANCE SHEET as at 30th September, 1934.

### LIABILITIES AND CAPITAL.

| | £ | s. | d. | £ | s. | d. |
|---|---|---|---|---|---|---|
| Sundry Creditors .. .. | | | | 7 | 6 | 8 |
| Capital as at 30th Sept., 1933 .. .. .. .. .. | 778 | 4 | 2 | | | |
| *Less* Deficit on Income and Expenditure Account for the year | 30 | 7 | 4 | | | |
| | | | | 747 | 16 | 10 |
| | | | | £755 | 3 | 6 |

### ASSETS.

| | £ | s. | d. | £ | s. | d. |
|---|---|---|---|---|---|---|
| Cash at Bank— | | | | | | |
| Current Account .. .. | 118 | 10 | 4 | | | |
| Deposit Account .. .. | 166 | 2 | 8 | | | |
| Cash in hands of Treasurer | 1 | 11 | 6 | | | |
| | | | | 286 | 4 | 6 |
| £500 4 per cent. Consolidated Stock at cost .. .. .. .. .. .. | | | | 437 | 9 | 0 |
| Sundry Debtors— Members' Subscriptions for 1933–1934 outstanding .. | | | | 31 | 10 | 0 |
| | | | | £755 | 3 | 6 |

Audited and found correct,

H. E. KEELER,

*Chartered Accountant,*

*Auditor.*

16–18 Clare Street, Bristol 1,

*9th October,* 1934

Dr. Charles spoke of the gratitude of the Society to Dr. George Parker for his work as Medical Librarian, and proposed that he should be re-elected, and the proposition was carried unanimously.

---

The second meeting of the session was held on Wednesday, 14th November, at 8.30 p.m., in the Physiological Lecture Theatre of the University.

Professor A. Rendle Short was in the Chair, and fifty-two members were present.

The minutes of the previous meeting were read and confirmed.

Dr. A. L. Taylor spoke of the Bristol Blood Transfusion Service, in the introduction of which he has taken such an active share.

Mr. Hamilton Bailey, F.R.C.S., then read a paper on "**The Diseases of the Testis,**" and this was illustrated by excellent lantern slides. Mr. Duncan Wood proposed and Mr. Chitty seconded a vote of thanks to Mr. Hamilton Bailey, and this was carried with acclamation.

---

### Extract from Editor's Report.

### COST OF THE "JOURNAL."

SOME of our readers who look at the Treasurer's accounts for the past year on opposite page may be alarmed that the cost of the *Journal* has exceeded the total of Members' Subscriptions. The expenditure on the *Journal* has been exceptional owing to the publication of a comprehensive Index to the first fifty volumes. This Index cost sixty-seven pounds, but members of the Society will agree that when a journal has lasted for half a century its value as a reference work is greatly enhanced by the issue of a Single Index containing the whole contents of the previous volumes in compact and accessible form.

